# Brain Transcriptome Analysis Links Deficiencies of Stress-Responsive Proteins to the Pathomechanism of Kii ALS/PDC

**DOI:** 10.3390/antiox9050423

**Published:** 2020-05-14

**Authors:** Satoru Morimoto, Mitsuru Ishikawa, Hirotaka Watanabe, Miho Isoda, Masaki Takao, Shiho Nakamura, Fumiko Ozawa, Yoshifumi Hirokawa, Shigeki Kuzuhara, Hideyuki Okano, Yasumasa Kokubo

**Affiliations:** 1 Department of Physiology, School of Medicine, Keio University, Tokyo 160-8582, Japan; satoru_morimoto@keio.jp (S.M.); ishimi@keio.jp (M.I.); hwatanabe@keio.jp (H.W.); miho_isoda_keio@yahoo.co.jp (M.I.); shiho.nakamura@keio.jp (S.N.); ozawa@z7.keio.jp (F.O.); 2 Department of Oncologic Pathology, School of Medicine, Mie University, Mie 514-8507, Japan; ultray2k@clin.medic.mie-u.ac.jp; 3 Department of Clinical Laboratory, National Center of Neurology and Psychiatry (NCNP), Tokyo 187-0031, Japan; msktakaobrb@ncnp.go.jp; 4 Graduate School of Health Science, Suzuka University of Medical Science, Mie 510-0293, Japan; kuzuhara@suzuka-u.ac.jp; 5 Kii ALS/PDC Research Center, Graduate School of Regional Innovation Studies, Mie University, Mie 514-8507, Japan

**Keywords:** amyotrophic lateral sclerosis (ALS), parkinsonism-dementia complex (PDC), Kii peninsula, transcriptome analysis, stress-responsive proteins, heat shock proteins, mitochondria, synaptic trafficking, HLA typing

## Abstract

Amyotrophic lateral sclerosis and Parkinsonism-dementia complex (ALS/PDC) is a unique endemic neurodegenerative disease, with high-incidence foci in Kii Peninsula, Japan. To gather new insights into the pathological mechanisms underlying Kii ALS/PDC, we performed transcriptome analyses of patient brains. We prepared frozen brains from three individuals without neurodegenerative diseases, three patients with Alzheimer’s disease, and 21 patients with Kii ALS/PDC, and then acquired microarray data from cerebral gray and white matter tissues. Microarray results revealed that expression levels of genes associated with heat shock proteins, DNA binding/damage, and senescence were significantly altered in patients with ALS/PDC compared with healthy individuals. The RNA expression pattern observed for ALS-type brains was similar to that of PDC-type brains. Additionally, pathway and network analyses indicated that the molecular mechanism underlying ALS/PDC may be associated with oxidative phosphorylation of mitochondria, ribosomes, and the synaptic vesicle cycle; in particular, upstream regulators of these mechanisms may be found in synapses and during synaptic trafficking. Furthermore, phenotypic differences between ALS-type and PDC-type were observed, based on HLA haplotypes. In conclusion, determining the relationship between stress-responsive proteins, synaptic dysfunction, and the pathogenesis of ALS/PDC in the Kii peninsula may provide new understanding of this mysterious disease.

## 1. Introduction

Amyotrophic lateral sclerosis (ALS) and Parkinsonism-dementia complex (PDC) (ALS/PDC) is a unique neurodegenerative disease, with high-incidence foci in Kii Peninsula (Japan), Guam (United States), and Western New Guinea (Indonesia) [1]. ALS and PDC in the Kii Peninsula (Kii ALS/PDC) tends to run in families and is characterized by abundant neurofibrillary tangles in the central nervous system [1,2,3,4]. Furthermore, Kii ALS/PDC is a multiple proteinopathy, presenting as a tauopathy similar to Alzheimer’s disease (AD), α-synucleinopathy similar to Parkinson’s disease (PD), and TAR DNA-binding protein 43 (TDP-43) proteinopathy similar to ALS [5,6]. Several hypotheses regarding pathogenic mechanisms underlying Kii ALS/PDC have been posited, including mineral and trace metal deficiencies, lifestyle changes, cycad toxin, and the neurotoxic β-methylamino-l-alanine (BMAA) theory [7], which are all based on the assumption that environmental factors play an important role in disease development. In addition, genetic factors have been examined [8]. Despite these efforts, the causes of Kii ALS/PDC remain unknown. Since completion of the Human Genome Project, transcriptomics has become a notable research field for the study of human diseases. Transcriptome analysis is a powerful tool that can reveal which RNAs are transcribed by the human genome in specific tissues under different environmental stimuli or specific pathological conditions [9].

In this study, we performed transcriptome analyses using Kii ALS/PDC brains to elucidate the underlying pathogenic mechanism of this disease.

## 2. Materials and Methods

This study was approved by ethics committees of Mie University (provided brain samples, Approval No. 2592), Keio University (Approval No. 20160273), and Mihara Memorial Hospital (provided brain samples, Approval No. 087-03).

### 2.1. Brain Samples

We prepared samples from the frozen brains of three healthy individuals (frontal lobe and temporal lobe, six total samples) as a control, three patients with sporadic AD (frontal lobe and temporal lobe, six total samples) as a tauopathy disease control, and 21 patients with Kii ALS/PDC (frontal lobe or temporal lobe). Profiles of samples are shown in Table 1. None of the patients had used drugs with clinical antioxidant activity at the time of autopsy.

### 2.2. Microarray Analysis

Total RNA was extracted using TRIzol Reagent (Thermo Fisher Scientific, Waltham, MA, USA), an RNase-Free DNase Set (Qiagen, Hilden, Germany), and an RNeasy Mini Kit (Qiagen). RNA quality was assessed using an Agilent 2100 Bioanalyzer (Agilent Technologies, Palo Alto, CA, USA). Values of 260/280 were more than 2.00 (mean ± SD = 2.11 ± 0.05) for all samples, and RNA Integrity Numbers of extracted RNA were as follows: 2.75 ± 0.42 for healthy control, 2.87 ± 0.48 for AD, and 3.93 ± 1.28 for Kii ALS/PDC. Total RNA (100 ng) was reverse transcribed, labeled with biotin using a TargetAmp-Nano Labeling kit (Epicentre, Madison, WI, USA), and hybridized to a HumanHT-12 v4 Expression BeadChip (Illumina, San Diego, CA, USA). Arrays were washed and stained with Cy3-Streptavidin, and then scanned with a BeadChip Scanner iScan System (Illumina), according to the manufacturer’s instructions.

Raw probe intensity data were normalized (RMA normalization at 85th percentile, low signal cutoff value of 100, base-2 log transformation, and mean ratio to control samples) using the transcriptome data analysis software Subio Platform (Subio, Kagoshima, Japan) (Supplementary Figure S1). Principal component analysis (PCA) was performed using normalized data. For hierarchical clustering, normalized data were calculated based on Euclidean correlations with average linkages. Analyses and visualizations of transcriptome data were performed using Subio Platform software. In addition, gene ontology (GO) analysis with the Database for Annotation, Visualization and Integrated discovery (DAVID, version 6.8), and pathway analysis with the Kyoto Encyclopedia of Genes and Genomes (KEGG) were performed. Visualizations of gene networks using the extracted gene set were performed using the GeneMANIA online tool (https://genemania.org/). Data included in this publication have been deposited into the NCBI Gene Expression Omnibus (GEO) database and are accessible with the GEO Series accession number GSE139384.

### 2.3. Quantitative RT-PCR Analysis

Total RNA was isolated with an RNeasy Mini Kit (Qiagen), with DNase I treatment, and cDNA was prepared using an iScript cDNA Synthesis Kit (Bio-Rad, Hercules, CA, USA). Quantitative reverse transcription polymerase chain reaction (qRT-PCR) was performed using 4 ng of cDNA per sample and SYBR Premix Ex Taq II (Takara Bio, Kusatsu, Japan) on a ViiA 7 Real-Time PCR System (Thermo Fisher Scientific). qRT-PCR primer details can be found in Supplementary Table S1. Relative expression values were calculated as follows: Ct = PCR cycle; ∆Ct = Ct (each target gene) − Ct (endogenous control, *ACTB*); ∆∆Ct = ∆Ct (each sample) − ∆Ct (calibrator, Control-1); and relative quantification = 2^−∆∆Ct^, in which Control-1 was set to 1.

### 2.4. Statistical Analysis

Statistical significance was calculated using Subio Platform software, DAVID, KEGG, and JMP11 (SAS Institute, Cary, NC, USA). Student’s *t*-test was used to compare fold changes, and a modified Fisher’s exact test was used for pathway/GO enrichment analyses. Wilcoxon rank sum test was used for qRT-PCR data. A likelihood-ratio chi-squared test was used for categorical data. *p*-values less than 0.05 were considered to demonstrate significant differences.

## 3. Results

### 3.1. Gene Expression Patterns in Kii ALS/PDC Forebrains

**Figure 1:**
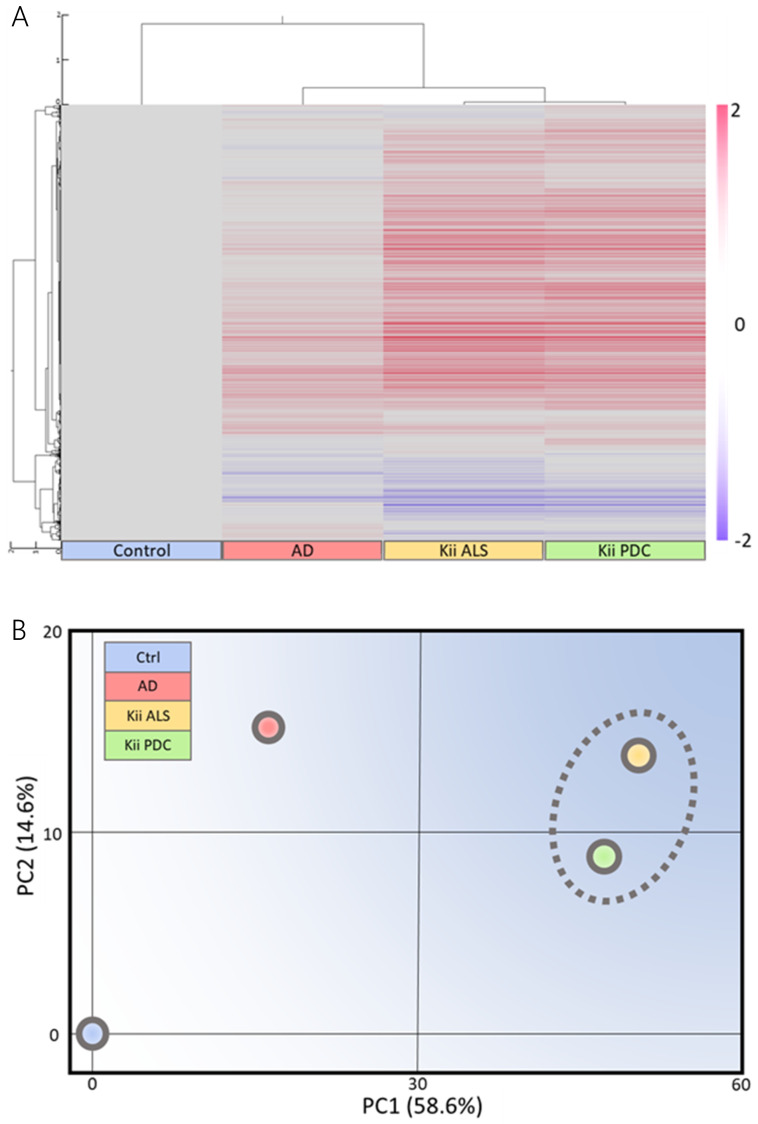

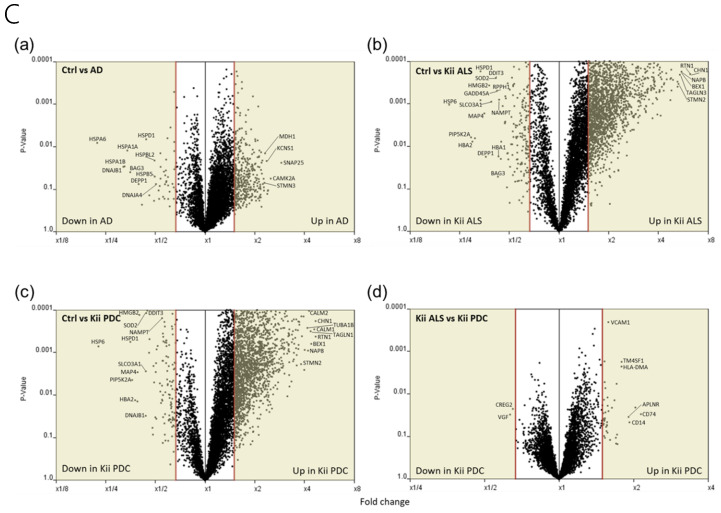
Microarray analysis of Kii amyotrophic lateral sclerosis and Parkinsonism-dementia complex (ALS/PDC) brains. (**A**) Clustering and (**B**) schematic principal component analysis (PCA) analysis based on control among mean control, Alzheimer’s disease, Kii ALS, and Kii PDC samples using Subio Platform. Locations of Kii ALS and Kii PDC samples are close; therefore, expression patterns between Kii ALS and Kii PDC are similar. (**C**) Volcano plots comparing control and (**a**) Alzheimer’s disease (AD), (**b**) Kii ALS, and (**c**) Kii PDC, and (**d**) between Kii ALS and Kii PDC. Cream colors indicate regions with differential gene expression greater than 1.5-fold. The number of differentially expressed genes was larger when comparing Kii ALS/PDC samples with control samples than when comparing AD samples with control samples.

**Figure 2:**
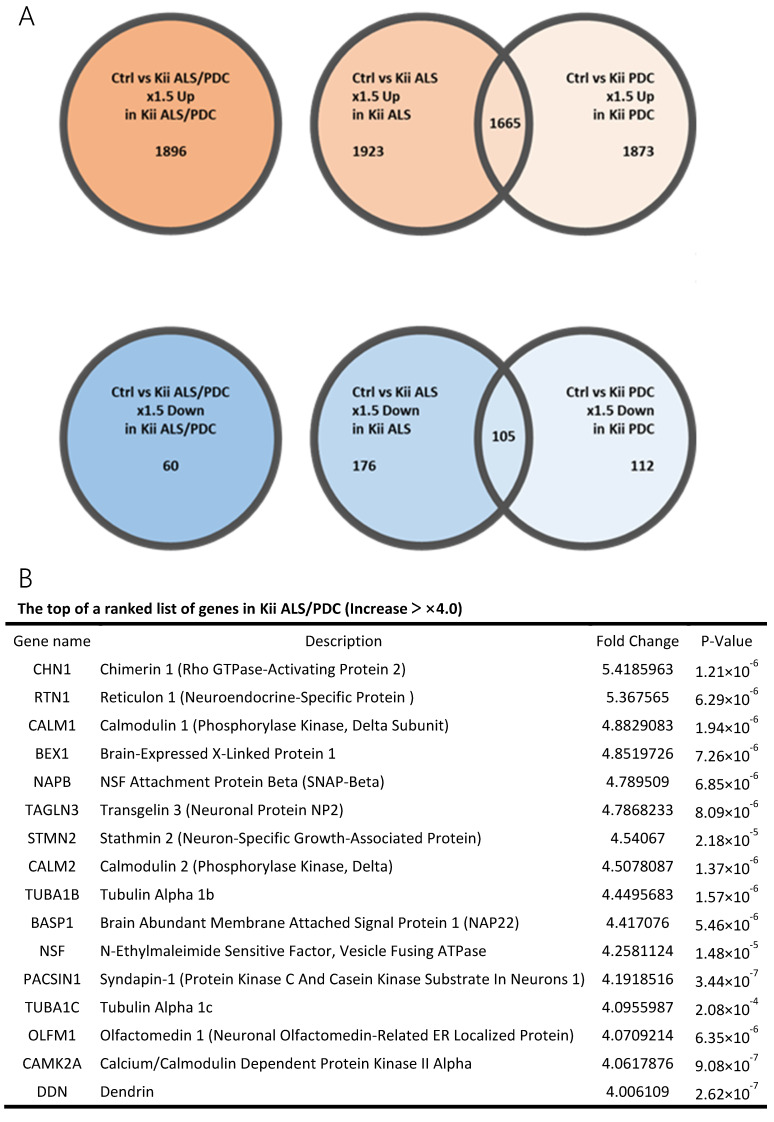

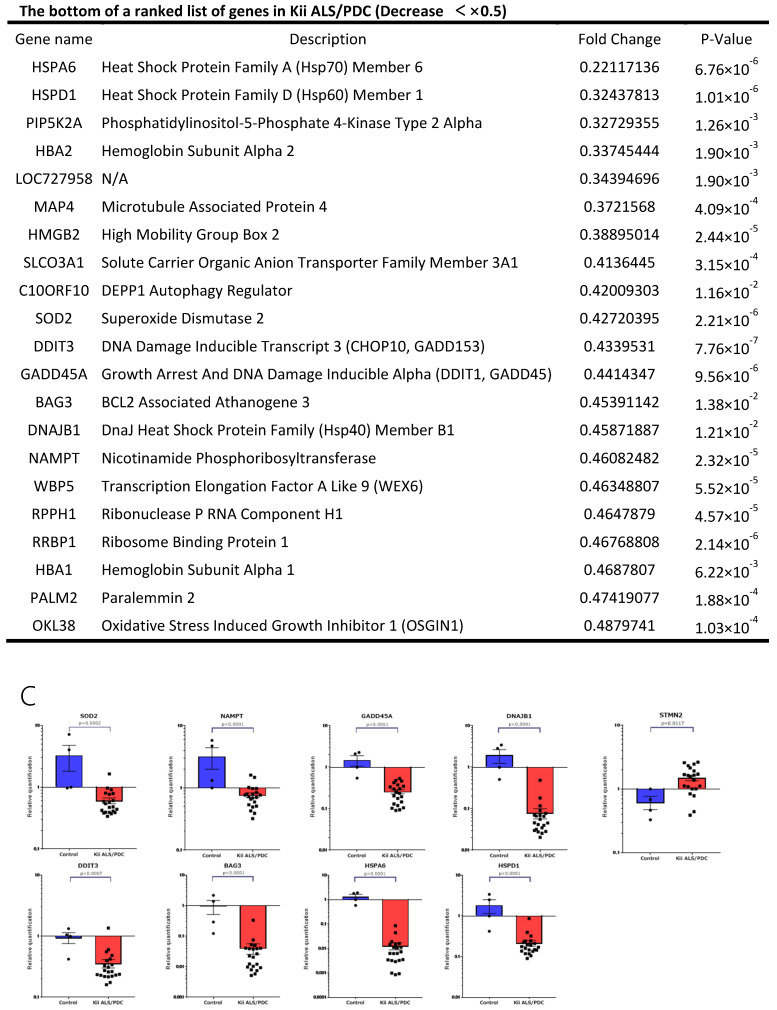
Genes with large fluctuations in Kii ALS/PDC brains and comparison of gene expression levels between control and Kii ALS/PDC samples. (**A**) Venn diagrams showing numbers of up- and downregulated genes among control, Kii ALS, Kii PDC, and Kii ALS/PDC samples. (**B**) The top and bottom of a ranked list of differentially expressed genes in Kii ALS/PDC samples compared with control samples. (**C**) qRT-PCR analysis of cardinal downregulated genes in Kii ALS/PDC samples. Expression levels of *SOD2*, *NAMPT*, *GADD45A*, *DNAJB1*, *DDIT3*, *BAG3*, *HSPA6*, and *HSPD1* were significantly decreased in all Kii ALS/PDC brain samples compared with controls (Control-1 and Control-3). The expression levels of *STMN2* increased significantly in all Kii ALS/PDC brain samples compared with those in controls (Control-1 and Control-3). Error bars indicate standard error of the mean (SEM). Relative quantification levels are displayed as a base-10 logarithm.

Clustering analysis and PCA showed that gene expression patterns in Kii ALS/PDC forebrains were different from those in healthy and AD brains, which were examined as a representative tauopathy. However, when we further divided ALS/PDC brains into Kii ALS and PDC subgroups, the expression patterns in Kii ALS brains were quite similar to those in Kii PDC brains, likely because Kii ALS and PDC represent components of the same disease entity (Figure 1). The original PCA data are shown in Supplementary Figure S2. Moreover, in the volcano plot, the number of differentially expressed genes between control and Kii ALS/PDC brains was larger than the number between control and AD brains (Figure 1C). Specifically, 1896 genes were upregulated by at least 1.5-fold in ALS/PDC brains compared with controls, whereas 60 genes were downregulated (Figure 2A). Furthermore, when subgroup analyses were performed, 1923 and 1873 genes were upregulated by at least 1.5-fold in Kii ALS and PDC brains compared with control brains, respectively, whereas 176 and 112 genes were downregulated, respectively. Most differentially expressed genes identified in Kii ALS and PDC brains compared with controls were common between the two subgroups (1665 and 105 common genes were upregulated and downregulated, respectively). The top (upregulated more than 4-fold) and bottom (downregulated more than 0.5-fold) differentially expressed genes were primarily associated with neurons, heat shock proteins (Hsps), DNA binding/damage, and senescence (Figure 2B). Moreover, we confirmed decreased expression levels of several genes related to various stress-responsive proteins, such as *superoxide dismutase 2* (*SOD2*), *nicotinamide phosphoribosyltransferase* (*NAMPT*), *DNA damage-inducible transcript 1* (*DDIT1*, also known as *GADD45A*), *DDIT3* (also known as *GADD153* and *CHOP10*, related to endoplasmic reticulum stress), *DNAJB1* (*HSP40*), *Bcl-2 associated athanogene 3* (*BAG3*), *Hsp family A member 6* (*HSPA6*), and *Hsp family D member 1* (*HSPD1*) by qRT-PCR analysis (Figure 2C).

### 3.2. GO Term Enrichment and KEGG Pathway Analysis of Kii ALS/PDC Forebrains

**Figure 3:**
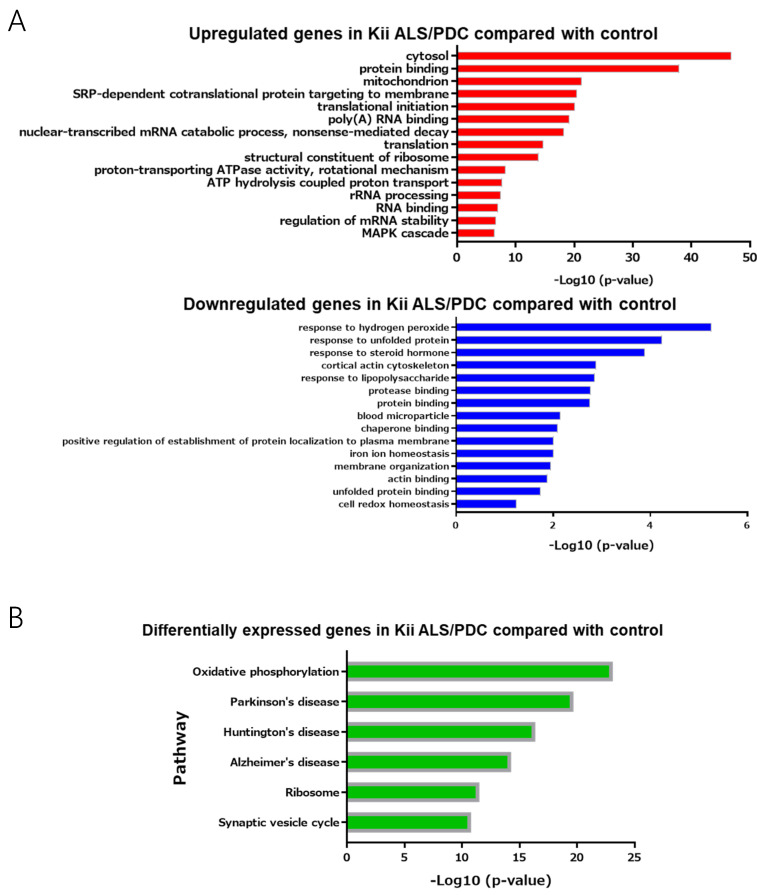

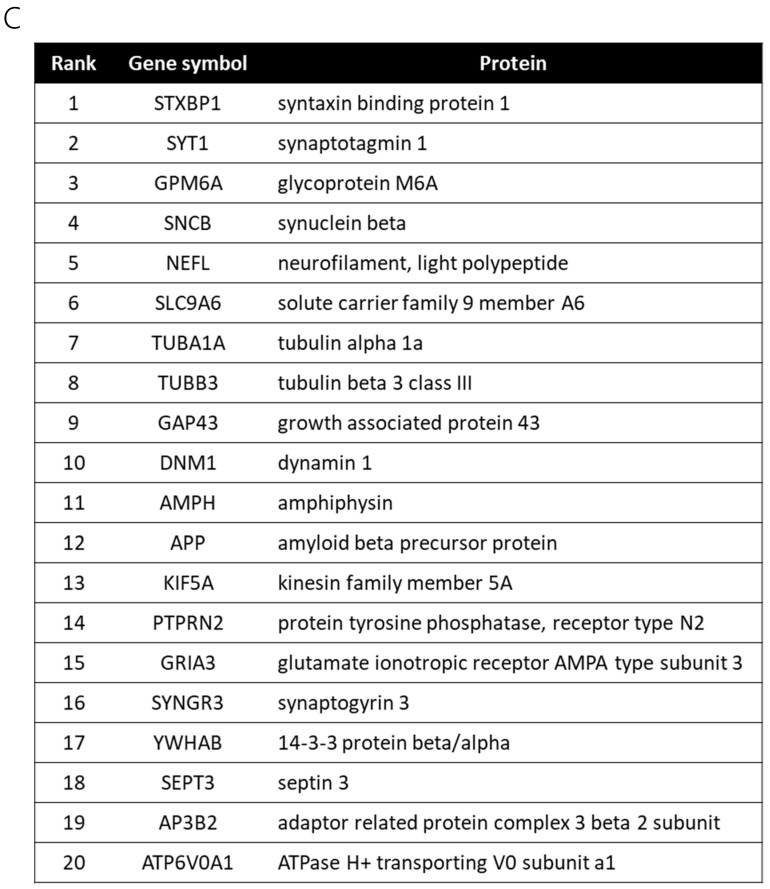
Gene ontology enrichment analysis and pathway analysis. (**A**) Up- or downregulated genes with fold-changes > 1.5 were analyzed by the Database for Annotation, Visualization and Integrated Discovery (DAVID, version 6.8) for gene ontology (GO) enrichment analysis. (**B**) Up- and downregulated genes with fold-changes > 1.5 were analyzed by Kyoto Encyclopedia of Genes and Genomes (KEGG) pathway enrichment analysis. (**C**) A network analysis of upregulated (2.50–5.42-fold) and downregulated (0.22–0.66-fold) genes between control and Kii ALS/PDC samples, as performed with GeneMANIA. The top 20 genes identified as being upstream of differentially expressed genes in Kii ALS/PDC samples compared with control samples.

GO analyses, using DAVID showed that upregulated genes identified in ALS/PDC brains were associated with the terms “RNA processing” and “mitogen-associated protein kinase (MAPK) cascade”, whereas downregulated genes were primarily associated with the terms “protein processing” and cell “redox homeostasis against oxidative stress” (Figure 3A). These results are in agreement with previously reported data [10,11].

In support of the above results, KEGG pathway enrichment revealed that the molecular mechanism underlying ALS/PDC may be related to oxidative phosphorylation of mitochondria, ribosomes, and the synaptic vesicle cycle. Moreover, pathogenic mechanisms and disease biomarkers associated with ALS/PDC may be similar to those for AD, PD, and Huntington’s disease (Figure 3B).

### 3.3. Gene Network Analysis of Kii ALS/PDC Forebrains

Thus far, we have shown relevance to biochemical signals and diseases, but have not yet been able to address specific gene functions. Therefore, we explored genes upstream of the identified differentially expressed genes (downregulated more than 0.66-fold or upregulated more than 2.5-fold) to reveal master regulators and biological processes associated with ALS/PDC, using GeneMANIA. Notably, the top 20 genes identified as being upstream of differentially expressed genes in ALS/PDC brains produced synapse and synaptic vesicle component proteins, including cytoskeletal proteins (especially those localized in presynaptic terminals) (Figure 3C).

### 3.4. Differences in Gene Expression between Kii ALS and Kii PDC, According to Phenotypic Classifications

Neuropathological analyses of lesions corresponding to the brain regions used for microarray analysis showed that the accumulation of disease-specific abnormal proteins (phosphorylated tau, *p* = 0.0355; phosphorylated α-synuclein, *p* = 0.0186; and phosphorylated TDP-43, *p* = 0.6624) in Kii PDC brains tended to be more severe than in Kii ALS brains (Supplementary Figure S3). In addition, several differences in gene expression levels (34 downregulated and two upregulated genes in Kii ALS compared with Kii PDC) were observed between Kii ALS and PDC brains (Supplementary Figure S4A). In particular, genes related to the human leukocyte antigen (HLA) family and glial cells, such as astrocytes, were identified by gene network analysis (Supplementary Figure S4B).

### 3.5. Categorization of Kii ALS/PDC by Clustering

Clustering analysis of gene expression in ALS/PDC brains revealed that they could be divided into two groups (A and B, Supplementary Figure S5). Upstream genes for all differentially expressed genes between groups A and B were explored using GeneMANIA, and the top 20 genes identified as being upstream of differentially expressed genes in ALS/PDC brains were ribosomal proteins (Supplementary Table S2).

## 4. Discussion

This study represents the first transcriptome analysis of 21 human brains from ALS/PDC patients. Kii ALS/PDC is a peculiar disease, presenting with multiple clinical phenotypes, such as motor neuron symptoms, Parkinsonism, dementia, and neuropathological multi-proteinopathies including tauopathy, α-synucleinopathy, and TDP-43 proteinopathy. In this study, we revealed that Kii ALS and PDC brains showed similar gene expression patterns, despite presenting with different clinical phenotypes (Figure 1).

Among genes expressed in ALS/PDC brains that differed significantly from control brains, we focused on Hsps. (Figure 2B). Hsps are ubiquitously expressed chaperone proteins that enable cells to cope with environmental stresses that cause protein misfolding. In mammalian cells, the protein quality control system becomes less effective with aging. In patients with neurodegenerative disorders, such as AD, PD, ALS, and ALS/PDC [12], the characteristic proteins associated with each disease (phosphorylated tau, phosphorylated α-synuclein, and phosphorylated TDP-43) are misfolded, which results in the formation of abnormal intracellular protein aggregates, leading to cellular dysfunction and, eventually, neuronal cell death. An important key for coping with misfolded proteins is the proteostasis mediated by Hsps, which act in protein folding, transport, and degradation, via proteasomal and autophagic pathways. Remarkably, the Hsp family is involved in all steps of proteostasis, including protein refolding, the unfolded protein response (UPR), protein degradation, mitophagy, and stress granule formation [13]. In our data, gene expression levels of Hsp family members *DNAJB1* (*HSP40 member)*, *HSPD1* (*HSP60 member)*, *HSPA6* (*HSP70 member)*, and *BAG3* were all significantly decreased in ALS/PDC brains, a finding that was further confirmed by qRT-PCR analysis (Figure 2C). HSP40 is involved in protein refolding and protein degradation through both proteasome and autophagy pathways, whereas the other member of HSP40, DNAJC7, was reported as a novel gene involved in ALS [14]. HSP60 plays a role in mitophagy and the mitochondrial UPR (mtUPR). HSP70, which is heavily involved in all steps of proteostasis, is a negative regulator of NLRP3 inflammasome activation [15], which drives tau pathology [16]. Thus, reduction of HSP70 may lead to the taupathology characteristics of ALS/PDC. BAG3 is essential for stress granule formation, which is cytoplasmic foci that form in response to many types of external stimuli and are essential to motor neurons survival following various stress in ALS. Therefore, decreased expression levels of *HSP* family members and *GADD* (a stress-responsive protein) in ALS/PDC brains is presumed to cause processing failures resulting in abnormal proteins (phosphorylated forms of tau, α-synuclein, and TDP-43), RNAs, and mitochondria.

In addition, oxidative stress is an important factor in the pathogenic mechanism underlying ALS/PDC [14]. SOD2 is a mitochondrial matrix enzyme that scavenges reactive oxygen species (ROS) produced by the extensive oxidation-reduction and electron transport reactions that occur in mitochondria. Decreases in *SOD2* expression lead to excessive mitochondrial production of ROS and mitochondrial dysfunction.

The onset of ALS/PDC is associated with aging, similar to many neurodegenerative diseases, and the relationship between aging and nicotinamide (vitamin B3, NAD) biology, including NAMPT, is notable [17]. NAMPT is an enzyme that converts NAD to nicotinamide mononucleotide (NAM), and is the rate-limiting component of the nicotinamide adenine dinucleotide (NAD+) biosynthesis pathway, which is crucial for mitochondrial homeostasis. Intracellular NAMPT (iNAMPT) protein levels are reportedly to be significantly reduced in the spinal cords of patients with ALS, indicating the involvement of NAMPT in pathophysiology of ALS [18]. Thus, the observed decrease in *NAMPT* expression in ALS/PDC brains suggests that abnormal mitochondrial bioenergetics may represent a key pathogenic mechanism underlying ALS/PDC.

Recently, Klim, et al. reported decreased *STMN2* expression after *TDP-43* knockdown and mislocalization in both patient-specific motor neurons and postmortem patient spinal cord issue [19]. However, *STMN2* expression was significantly upregulated in mouse lower motor neurons with *SOD1* mutation [20] and human neural progenitors derived from sporadic AD patient induced pluripotent stem cells (iPSCs) with high *DCX* and *ASCL1* expression [21]. Notably, our results indicated significantly increased expression of *STMN2* in ALS/PDC brains, including cases with cytoplasmic phosphorylated TDP-43 accumulation (Figure 2C). Thus, STMN2 expression may be differently regulated between ALS and Kii ALS/PDC, or between cortical and motor neurons.

Previously reported ALS causative genes have been associated with abnormal RNA processing, endosomal trafficking, autophagy, the endoplasmic reticulum, membrane, cytoskeleton, and mitochondria [22,23]. PD-causative genes have been demonstrated to have molecular mechanisms similar to those of ALS-causative genes [24]. Importantly, the synaptic membrane and endosomal trafficking pathways are common to both ALS and PD, whereas RNA trafficking is emphasized in ALS and mitochondrial dysfunction is the prevailing mechanism in PD. Furthermore, oxidative stress and nitrative stress have been implicated in the pathogenesis of ALS/PDC [10,11].

In this study, pathway and network analyses (Figure 3) indicated that the molecular mechanism underlying ALS/PDC may be related to oxidative phosphorylation of mitochondria, ribosomes, and the synaptic vesicle cycle; in particular, upstream regulators associated with the pathogenic mechanism may be present in synapses and involved in synaptic trafficking. Therefore, our results imply a pathomechanical aspect of synaptopathy in ALS/PDC. With regard to neurodegenerative diseases, the term synaptopathy was first used to explain the pathogenic mechanism underlying Huntington’s disease, and was later extended to the pathogenic mechanisms underlying AD [25], PD [26], and ALS [27]. Therefore, the clinical and neuropathological commonality among Kii ALS/PDC, AD, PD, and ALS may be derived from synaptic dysfunctions. Interestingly, identified upstream regulators included β-synuclein. α-Synuclein, which is accumulated in the central nervous system of Kii ALS/PDC patients, can bind several RNAs and DNAs or interact with histones, thereby regulating expression of various genes [28]. For example, overexpression of α-synuclein prompts mono and dimethylation of histone H3K9, leading to an increase of the methylated form of this histone at the SNAP25 promoter, almost certainly preventing SNARE complex assembly and fusion of synaptic vesicles [29]. Additionally, α-synuclein binds the 26S proteasome subunit (S6ʹ), and the binding of aggregated α-synuclein may suppress proteasome activity by sterically inhibiting substrate access to the proteasome. In contrast, β-synuclein prevents this interaction with S6ʹ by binding α-synuclein. As a result, β-synuclein prevents the binding of aggregated α-synuclein to the proteasome, thus antagonizing the stagnation of aggregated α-synuclein on the 26S proteasome [30].

In clustering analysis, we were able to classify ALS/PDC cases into two groups, according to gene expression patterns. Surprisingly, the results of exploratory network analyses of upstream factors strongly implied the involvement of ribosomal proteins in group (A) (Supplementary Table S2). A previous study revealed the destabilization of RNAs encoding oxidative phosphorylation and ribosomal components in *C9orf72*-linked familial ALS fibroblasts and iPSCs and in control iPSCs overexpressing *TDP-43* [31]. Reduced levels of oxidative phosphorylation and ribosomal transcripts were also detected in the spinal cords and brains of ALS and frontotemporal dementia (FTD) patients. These findings in fibroblasts, iPSCs, and human autopsy samples strongly implicate abnormalities in oxidative phosphorylation and ribosomal pathways in ALS and FTD cases characterized by TDP-43 pathology. Ribosomes are also important for local RNA translation, after transport by FUS and TDP-43, and for repeat-associated non-ATG (RAN) translation in C9orf72-linked familial ALS [32]. Ribosomal RNA (rRNA), the most abundant non-coding RNA, is a target for oxidative nucleobase damage by ROS [33]. Oxidized 23S rRNA inhibits ribosomes during protein biosynthesis. The placement of single oxidized nucleobases at specific positions within the catalytic center of ribosomes using atomic mutagenesis resulted in significantly different functional outcomes [33]. Therefore, abnormal ribosomal proteins and translation may be associated with the pathogenic mechanism for a subgroup of Kii ALS/PDC patients.

Moreover, the results of gene network analyses in this study suggest that differences in clinical phenotypes of Kii ALS and PDC in members of the same family may be associated with the HLA protein family. Previous reports showed that the HLA-DRA/HLA-DRB5 polymorphism affects the risk of developing sporadic ALS [34], while the loss of major histocompatibility complex (MHC) class 1 (corresponding to HLA in human beings) in motor neurons increased their vulnerability to astrocyte-mediated toxicity [35]. In addition, PD has been associated with HLA-DRB5*01 and HLA-DRB1*15:01 MHC alleles, and the T cells of PD patients recognized α-synuclein peptides via two MHC class II beta chain alleles: HLA-DRB5*01:01 and HLA-DRB1*15:01. Moreover, HLA-DRB5-DRB1 and HLA-A*03:01/HLA-B*07:02/HLA-DRB1*15:01/HLA-DQA1*01:02/HLA-DQB1*06:02 haplotypes are reportedly risk factors for AD. Therefore, HLA haplotypes may also influence phenotypic differences in ALS/PDC.

Kii ALS/PDC displays characteristic glial pathology, such as gliosis and the accumulation of phosphorylated tau in astrocytes and Bergmann glia [3]. Therefore, it is important to consider differences in the activities and functions of glial cells between Kii ALS and PDC, including the results of this study. The brain contains various cell types; however, this experiment did not allow the determination of which cell types were affected. In the future, cell-specific analyses will be necessary to revalidate our results, including cell sorting, nuclear sorting, and research using iPSCs.

Based on our analysis of patient brain transcriptomes, we hypothesize that the following modalities could have therapeutic potential: (1) inducing Hsps [36]; (2) increasing proteasome levels or activity, such as through agonist-induced conformational alteration, Nrf2 activation, or modulation of posttranslational modification using small molecules [37]; (3) reducing oxidative stress [38]; and (4) boosting NAD+ with small molecule NAMPT activators such as SBI-797812 [39].

## 5. Conclusions

In conclusion, we identified a potential component of the pathogenic mechanism underlying Kii ALS/PDC using transcriptome analyses of human brain samples. This study is the first to describe transcriptome analyses of ALS/PDC patients, although transcriptome analyses of other Western Pacific ALS/PDC patient populations, such as those from Guam and West New Guinea, remain to be performed. The results of this study represent an important step toward overcoming this intractable disease, for which no useful treatments currently exist. Our findings identify potential new targets associated with abnormalities in the protein quality control system, such as stress-responsive proteins, synapse-related defects, and mitochondrial dysfunction. However, future research should verify whether deficiencies of stress-responsive proteins are causes or symptoms of this disease. The results of this study should contribute greatly to revealing the causes of Kii ALS/PDC.

## Figures and Tables

**Table 1:** Profiles of brain samples.

Case	Gender	Age at Death	Phenotype	Sample
**Control-1**	Male	75	-	Frontal lobe
				Temporal lobe
**Control-2**	Male	83	-	Frontal lobe
				Temporal lobe
**Control-3**	Male	87	-	Frontal lobe
				Temporal lobe
**AD-1**	Male	67	D	Frontal lobe
				Temporal lobe
**AD-2**	Male	86	D	Frontal lobe
				Temporal lobe
**AD-3**	Male	74	D	Frontal lobe
				Temporal lobe
**Kii-1**	Female	66	ALS	Frontal lobe
**Kii-2**	Male	77	ALS + D	Frontal lobe
**Kii-3**	Female	70	PDC + A	Frontal lobe
**Kii-4**	Female	74	ALS	Frontal lobe
**Kii-5**	Female	76	PDC + A	Frontal lobe
**Kii-6**	Female	60	PDC + A	Temporal lobe
**Kii-7**	Male	79	PDC + A	Frontal lobe
**Kii-8**	Female	71	PDC + A	Frontal lobe
**Kii-9**	Female	63	ALS	Frontal lobe
**Kii-10**	Male	65	ALS + D	Frontal lobe
**Kii-11**	Female	70	ALS	Frontal lobe
**Kii-12**	Female	81	ALS	Frontal lobe
**Kii-13**	Female	70	PDC	Frontal lobe
**Kii-14**	Male	74	PDC	Frontal lobe
**Kii-15**	Female	73	ALS	Frontal lobe
**Kii-16**	Male	72	ALS + D	Frontal lobe
**Kii-17**	Female	72	PDC	Temporal lobe
**Kii-18**	Male	75	PDC	Frontal lobe
**Kii-19**	Male	85	PDC	Frontal lobe
**Kii-20**	Female	76	ALS	Frontal lobe
**Kii-21**	Female	74	PDC	Temporal lobe

Abbreviation: A; amyotrophy, AD; Alzheimer’s disease, ALS; amyotrophic lateral sclerosis, D; dementia, PDC; Parkinsonism-dementia complex.

## Supplementary Materials

The following are available online at https://www.mdpi.com/2076-3921/9/5/423/s1, Figure S1: Histograms showing densities of log-intensities after global normalization using the Subio Platform. Figure S2: Principal component analysis (PCA) of all brains. Figure S3: Clustering tree of all cases and clinical/neuropathological profiles of KiiALS/PDC patients. Figure S4: Network analysis between Kii ALS and PDC. Figure S5: Classification of KiiALS/PDC samples, based on clustering tree and principal component analysis (PCA). Table S1: List of qRT-PCR primers. Table S2: Genes located upstream of differentially expressed genes between KiiALS and PDC.
