# Supplementary material for: Brain Transcriptome Analysis Links Deficiencies of Stress-Responsive Proteins to the Pathomechanism of Kii ALS/PDC
Source: Antioxidants (Basel). 2020 May 14;9(5):423. doi: 10.3390/antiox9050423 (PMC7278732; doi:10.3390/antiox9050423)
Supplement: Supplementary file 1 [file antioxidants-09-00423-s001.pdf]

Supplementary Figure 1

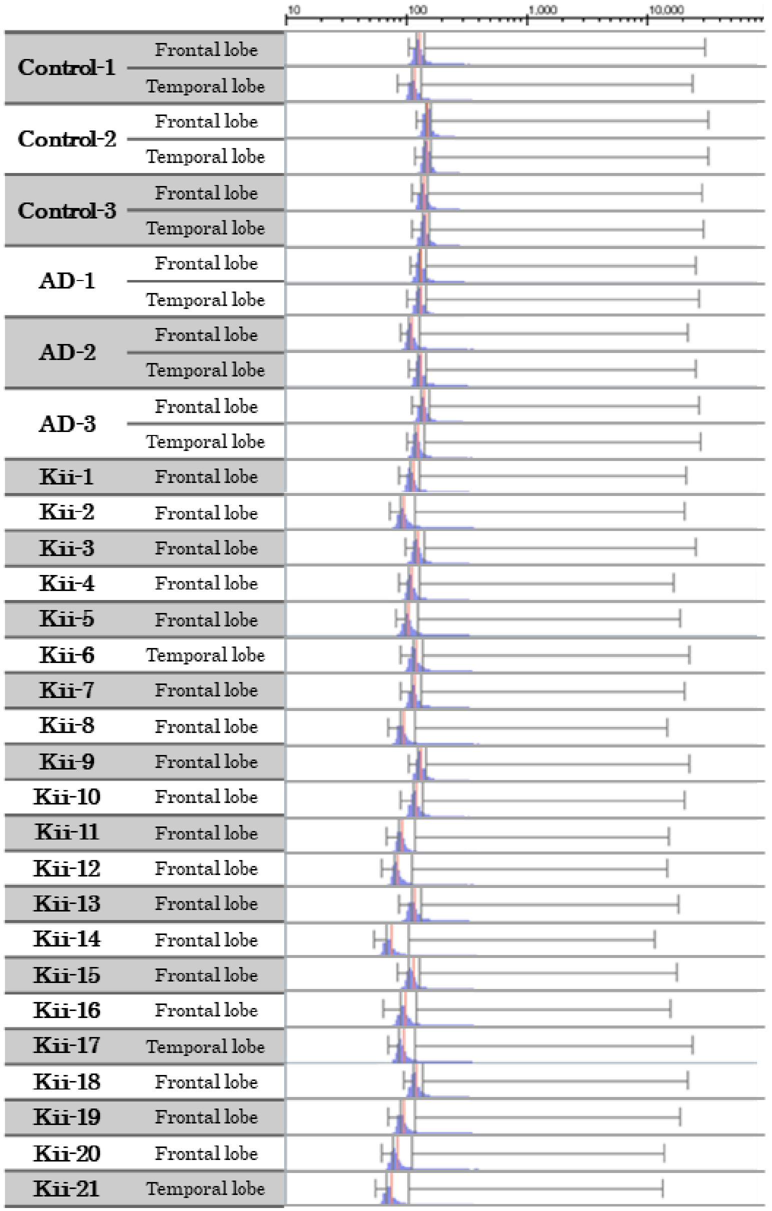

Figure legend. The histograms show the densities of log-intensities after global normalization, using the Subio platform.

Supplementary Figure 2

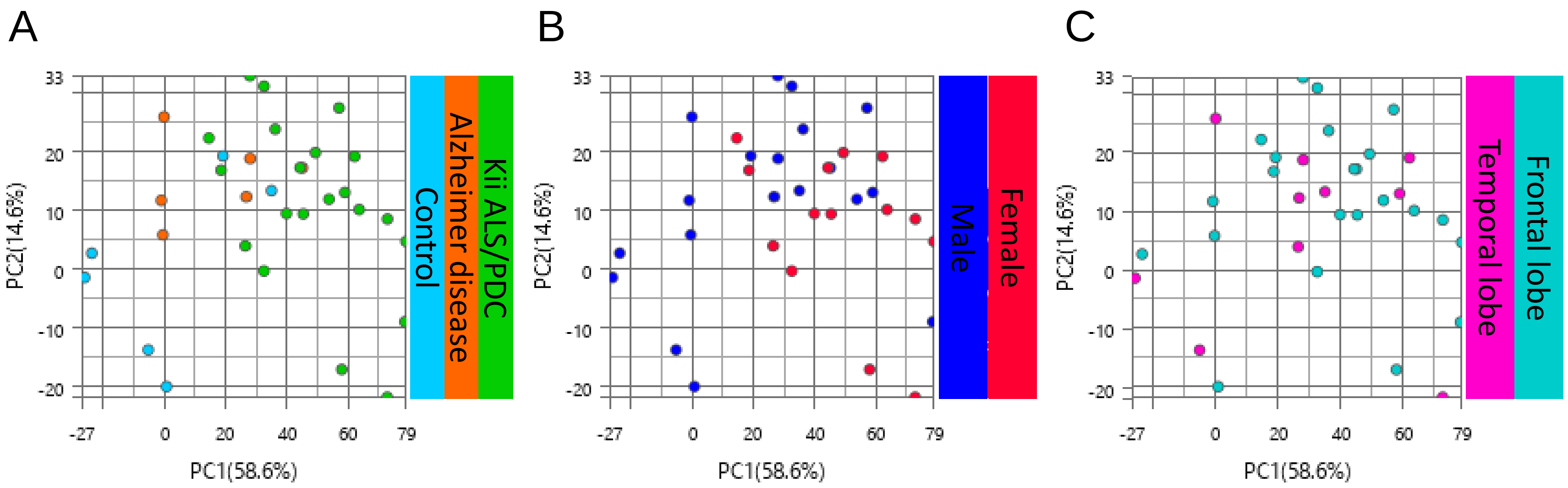

Figure legend. Primary component analysis (PCA) (A) among control, Alzheimer disease, Kii ALS, and Kii PDC samples, (B) between male and female, and (C) between temporal lobe and frontal lobe, using the Subio platform.

Supplementary Figure 3

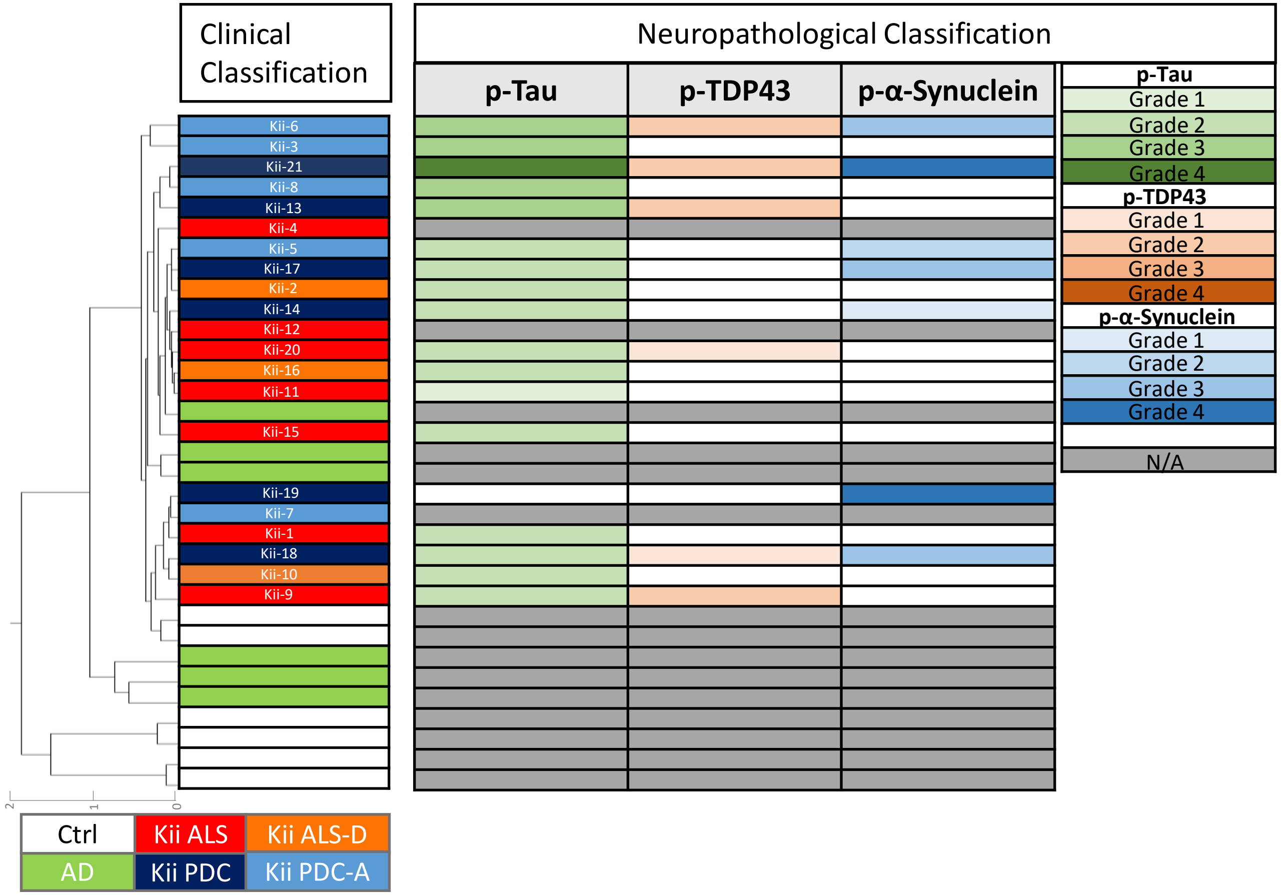

Figure legend. Clustering tree of all cases, using Subio Platform, and the clinical/neuropathological profiles of Kii ALS/PDC patients. Neuropathological profiles in the brain regions submitted for microarray analysis are shown.

Supplementary Figure 4

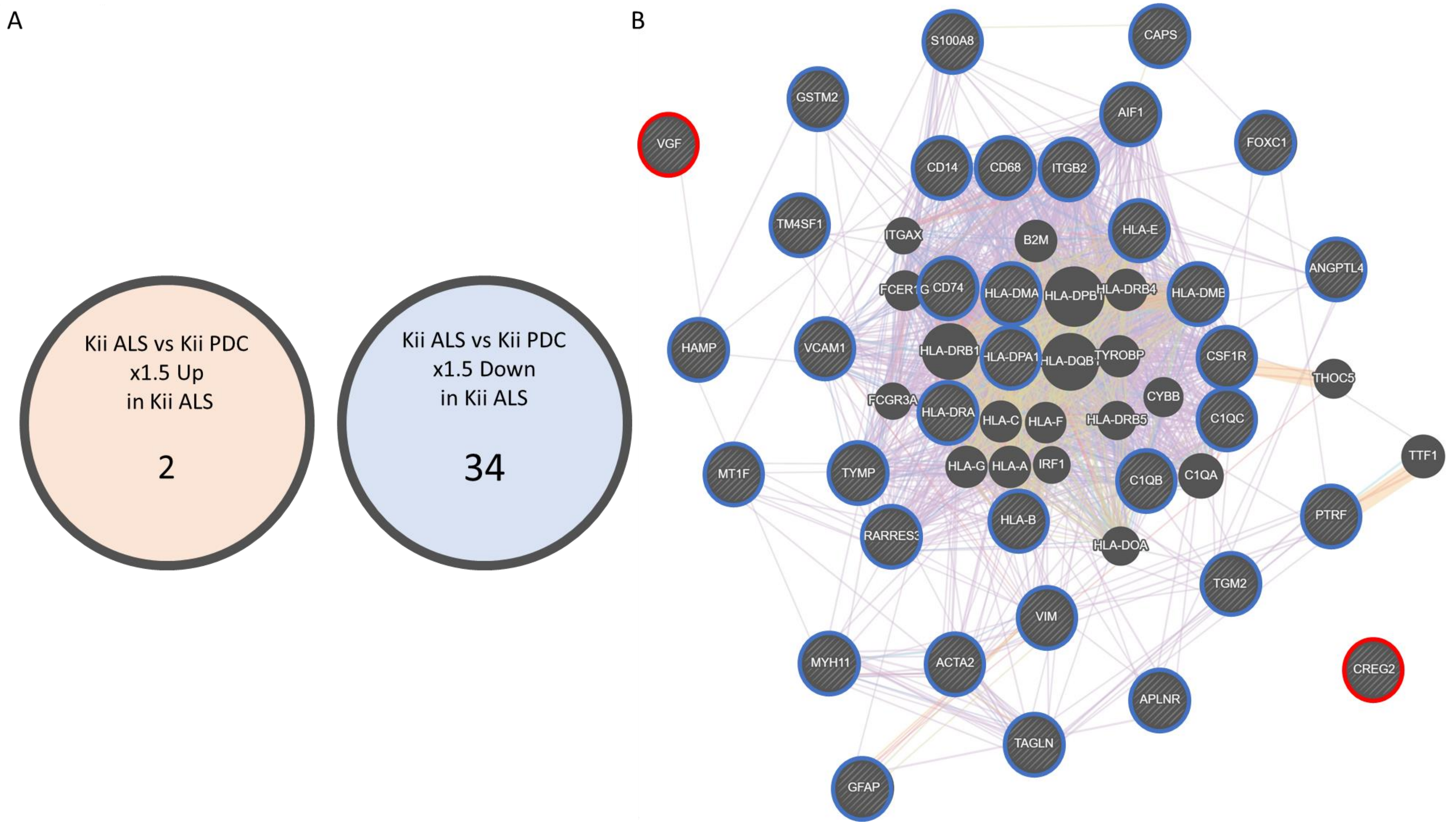

Figure legend. A network analysis by GeneMANIA. (A) The number of differentially expressed genes, using Subio Platform and (B) a network analysis by GeneMANIA of the upregulated (fold-change > 1.5) and downregulated (fold-change < 1.5) genes in Kii ALS compared with Kii PDC. Red circles indicate upregulated genes and blue circles indicate downregulated genes. The details of the illustration in (B) refer to https://genemania.org/.

Supplementary Figure 5

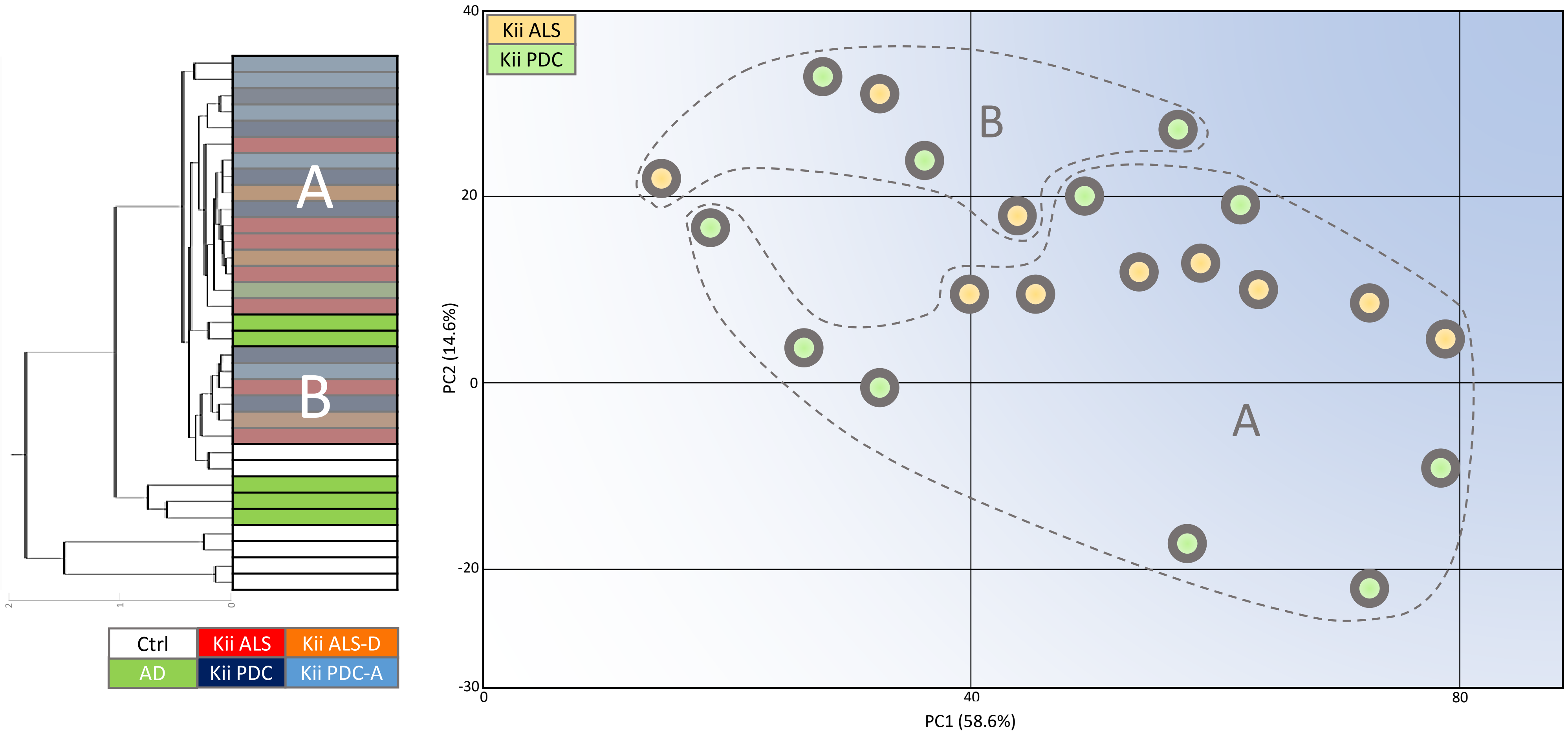

Figure legend. Classification of Kii ALS/PDC samples, based on clustering tree and primary component analysis. Kii ALS/PDC cases were divided into groups A and B.

Supplementary Table 1

Supplementary Table 1. List of qRT-PCR primers

Gene	Amplicon Size (bp)	Forward	Tm (℃)	Reverse	Tm (℃)	The Massachusetts General Hospital PrimerBank ID
ACTB	151	TGAAGTGTGACGTGGACATC	57.9	GGAGGAGCAATGATCTTGAT	54.9	NA<br>[Ref] Andoh-Noda T, et al. Mol Brain 2015;8:31.
SOD2	109	TTTCAATAAGGAACGGGGACAC	60.2	GTGCTCCCACACATCAATCC	60.7	67782304c3
NAMPT	172	ATCCTGTTCCAGGCTATTCTGT	60.6	CCCCATATTTTCTCACACGCAT	60.7	111161293c3
GADD45A	87	GAGAGCAGAAGACCGAAAGGA	61.2	CAGTGATCGTGCGCTGACT	62.4	9790904b1
DNAJB1	75	AAGGCATGGACATTGATGACC	60.4	GGCCAAAGTTCACGTTGGT	60.8	5453689c1
DDIT3	80	GAACGGCTCAAGCAGGAAATC	61.6	TTCACCATTCGGTCAATCAGAG	60.0	304282228c2
BAG3	112	ATTCCGGTGATACACGAGCAG	61.9	GCTGGTGGGTCTGGTACTC	61.4	62530382c3
HSPA6	115	GATGTGTCGGTTCTCTCCATTG	60.7	CTTCCATGAAGTGGTTCACGA	60.0	42822885c3
HSPD1	99	CTACTGTACTGGCACGCTCTA	60.7	CAACAGCTAACATCACACCTCTC	60.9	41399283c3
STMN2	84	GCTCTTGCTTTTACCCGGAAC	61.5	AGGCACGTTTGTTGATTTGCT	61.3	312922387c1

Supplementary Table 2

Supplementary Table 2. The genes located upstream of the differentially expressed genes between Kii ALS and Kii PDC, as analyzed by GeneMANIA

Rank	Gene symbol	Protein
1	RPL6	ribosomal protein L6
2	RPS23	ribosomal protein S23
3	RPS27A	ribosomal protein S27a
4	RPL7	ribosomal protein L7
5	RPL24	ribosomal protein L24
6	RPL11	ribosomal protein L11
7	RPS6	ribosomal protein S6
8	RPS25	ribosomal protein S25
9	RPL27	ribosomal protein L27
10	RPL10A	ribosomal protein L10a
11	RPL31	ribosomal protein L31
12	RPL34	ribosomal protein L34
13	RPS27	ribosomal protein S27
14	RPS17	ribosomal protein S17
15	RPS5	ribosomal protein S5
16	RPS29	ribosomal protein S29
17	RPLP0	ribosomal protein lateral stalk subunit P0
18	RPL32	ribosomal protein L32
19	RPS18	ribosomal protein S18
20	RPS24	ribosomal protein S24
